# The Depression Anxiety Stress Scale 8: investigating its cutoff scores in relevance to loneliness and burnout among dementia family caregivers

**DOI:** 10.1038/s41598-024-60127-1

**Published:** 2024-06-06

**Authors:** Amira Mohammed Ali, Abdulmajeed A. Alkhamees, Souheil Hallit, Tariq N. Al-Dwaikat, Haitham Khatatbeh, Saeed A. Al-Dossary

**Affiliations:** 1 Department of Psychiatric Nursing and Mental Health, Faculty of Nursing, Alexandria University, Smouha, 21527 Alexandria Egypt; 2 Department of Psychiatry, College of Medicine, Qassim University, Buraidah, Saudi Arabia; 3 School of Medicine and Medical Sciences, Holy Spirit University of Kaslik, P.O. Box 446, Jounieh, Lebanon; 4 Applied Science Research Center, Applied Science Private University, Amman, Jordan; 5 Department of Community and Mental Health, Faculty of Nursing, Jordan University of Science and Technology, Irbid, Jordan; 6 Department of Nursing, Faculty of Nursing, Jerash University, Jerash, Jordan; 7 Department of Psychology, College of Education, University of Ha’il, 1818 Ha’il, Saudi Arabia

**Keywords:** Mental health, Depression, Anxiety, Psychology, Diseases, Health care, Medical research, Risk factors, Signs and symptoms

## Abstract

The global trend of advanced aging comes at the cost of amplified onset of age-related diseases. Dementia is a common multifactorial age-related neurodegenerative disorder, which manifests with progressive declines in cognitive functioning and ability to perform activities of daily living. As polices discourage institutionalized care, family members act as primary caregivers and endure increased vulnerability to physical and mental health problems secondary to care-related changes in life routine and relationships. Targeting clinically significant distress at earlier stages through valid brief measures may promote caregivers’ wellbeing and dementia care continuity/quality. This study aimed to determine the optimal cutoff score of the Depression Anxiety Stress Scale 8-items (DASS-8) in a convenience sample of 571 European caregivers (Mean age = 53 ± 12 years, Italian = 74.4%, Swiss = 25.6%) through three methods. K-means clustering classified the sample into high- and low-distress clusters based on DASS-8 score of 19. Receiver operator curve (ROC) analysis using 48 and 7 cutoffs of the Zarit Burden Interview (ZBI) and the Three-Item University of California, Los Angeles, Loneliness Scale-version 3 (UCLALS3), revealed two DASS-8 cutoffs (12.5 and 14.5, area under the curve (AUC) = 0.85 and 0.92, p values < .001, 95% CI 0.82–0.88 and 0.89 to 0.94, sensitivity = 0.81 and 0.78, specificity = 0.76 and 0.89, Youden index = 0.57 and 0.67, respectively). Decision modeling produced two DASS-8 cutoffs (9.5 and 14.5) for predicting low and high caregiving burden and loneliness, respectively. According to the median of all DASS-8 cutoffs (14.5) the prevalence of mental distress was 50.8%. Distress correlated with key mental problems such as burnout and loneliness—in path analysis, DASS-8 scores were predicted by the ZBI, UCLALS3, care dependency, and receiving help with care, especially among older, female, and spouse caregivers. Further diagnostic workup should follow to confirm psycho-pathogenicity among caregivers with DASS-8 scores above 14.5. Investigations of the DASS-8 in other countries/populations may confirm the validity of this cutoff score.

## Introduction

By 2050, the global populations aged 65 and 80 years, in order, are expected to double and triple^1,2^. This unprecedented increase in life expectancy comes at the cost of amplified onset of age-related physical and mental disorders, with increasing trends toward the use of institutionalized care^3^. Dementia is a common age-related neurodegenerative disease, in which patients display progressive declines in cognitive functions and ability to perform activities of daily living. Because of increased risk factors (e.g., aging, lifestyle, loneliness, pollution, etc.), dementia incidence has considerably increased, and it is expected to reach 1.7 million cases in the UK by 2040^1,4^. Older adults benefiting from nursing homes witness a twofold increase in mortality relative to counterparts living in the community^2^. Therefore, policy makers’ top priority is to promote older people’ s capacity to live in the community and receive advanced health care services at home^3^.

Dementia care is primarily provided by family members, with more than 41 million Americans acting as informal caregivers^5^. Dementia family caregivers struggle with the deteriorating and demanding disease of their relatives^6^. Physical, psychological, relational, and financial burdens endured by caregivers put them at an increased risk for psychopathology^7–9^. Around two thirds dementia caregivers experience anxiety and depression symptoms^10,11^. Emotional difficulties in this population are associated with numerous challenges including behavioral symptoms, long duration of being caregivers, financial constraints, lack of knowledge, communication difficulties, lack of public awareness, concerns about the selection of care facilities, providing care and decision-making, and death with dignity^9,11,12^. Mental distress in dementia caregivers is associated with greater levels of care burden, poor health-related quality of life, and lower resilience^10,13^.

Caregiving burden and distress symptoms are at their highest among those facing high-care demand^10,13,14^, with increased vulnerability to social isolation and loneliness among individuals spending more time in caregiving activities^15,16^. Loneliness is a threatening chronic source of stress, which depletes physiological buffer mechanisms such as the internal antioxidant system, resulting in accelerated oxidative and inflammatory signaling (e.g., increased production of H_2_O_2_, interleukin-6, and tumor necrosis factor-alpha)^16,17^. Free radicals and inflammatory cytokines trigger generalized cellular degeneration, resulting in cognitive decline, pain, fatigue, depression, anxiety, and multiple physical health problems^5,17^.

Physical and mental problems potentiate caregiving burden and loneliness as caregivers attempt to meet their own life responsibilities and struggle with changes in daily routine associated with prolonged caregiving^5,15^. Despite the enormous challenges they encounter, up to 43% of family caregivers display resilience—high levels of psychological well-being noted by ability to resist and adapt to adversities (high caregiving demands) through a range of personal strengths/assets and environmental resources), eventually leading to positive outcomes^13^. Particularly, positive aspects of caregiving may buffer the effect of behavioral bother on caregivers’ symptoms of depression and anxiety^18^. Indeed, activities focused on enhancing the coping resources of caregivers by interrupting the vicious cycle of distress/burden/loneliness (e.g., support groups) may improve their quality of life and mental wellbeing, reduce loneliness, and boost their ability to provide better care for their patients^12,16,19^. Thus, identifying caregivers with greater proneness to distress is pivotal for interventions that target caregiver's own intra-psychic resources, coping, and resilience to be more effective in lowering psychiatric comorbidity, which may have implications for dementia care quality and continuity^10^.

Using a sample of students, Lovibond and Lovibond developed the Depression Anxiety Stress Scale (DASS, 1995) as a 42-item, three-dimensional measure of three interrelated negative emotional states (depression, anxiety and tension/stress), which comprise the tripartite model of distress^20^. Despite its high reliability and concurrent validity (correlating with parallel measures e.g., Beck Depression Inventory and Beck Anxiety Inventory)^20–22^, investigations in clinical and nonclinical samples revealed structural issues with the DASS. Aiming to produce a cleaner factor structure and smaller inter-factor correlations, scale developers generated the DASS-21 by reducing the number of items by half^20^. Taking advantage of its small number of items, this short form has been frequently used in research and clinical practice to identify people with common mental disorders (anxiety and/or depression)^20,21,23^. Meta-analyses generally support the original three-factor structure of both DASS measures, but bifactor models show superior fit in most studies—suggesting presence of an overall negative emotion factor, denoting that a unidimensional score of the DASS may account for the overlap between depression and anxiety^23,24^. Additionally, the DASS-21 records inconsistent results on core psychometric properties, including invariance across genders, responsiveness, and ceiling effects^23,25,26^, especially when used among adolescents and children. Indeed, Rasch analysis shows that the DASS-21 features disordered categories with multiple redundant items that assess traits at analogous levels among youth^27,28^.

Poor response rate is common among people with mental problems^29,30^. Therefore, shorter scales may be favorable for eliciting higher response rates in large-scale screening^31,32^. Accordingly, several researchers attempted to develop briefer versions to overcome the structural shortcomings of the DASS-21 (e.g., 18-, 17-, 14-, 13-, 12-, and 9-item DASS)^21,22,33–35^, with a model developed especially for young groups, the DASS-Youth^28^. Studies replicating these shortened models are lacking, which limits the practicality of their use in research or clinical settings.

The DASS-8 has been introduced in the early period of the COVID-19 pandemic by a group of Arab and Japanese researchers during the testing of various structures of the DASS-21 and its shortened versions in three samples: individuals suspected of or diagnosed with COVID-19 in quarantine facilities, psychiatric outpatients, and community-dwelling adults. Items retained were those demonstrating the highest frequency of occurrence and greatest values of item-total correlations. The DASS-8 retained the three-dimensional structure of the parent DASS-21 but expressed best fit and invariance across age and gender groups^26^. However, the inherently unique nature of distress during the early stage of the pandemic may render scale capacity for symptom detection inaccurate in subsequent stages as well as in the general population. Accordingly, the DASS-8 was further investigated in studies comprising samples collected before the pandemic from Gana, Australia, the UK, the US, as well as samples collected during the peak of the pandemic from Italy and Switzerland^36–38^. It expressed psychometric adequacy relative to the parent scale and a 12-item form of the DASS-21 (DASS-12) regarding its factor structure, measurement invariance, convergent validity, criterion validity, discriminant validity, and reliability^26,36–38^. Principally, it differentiated women with pelvic pain and caregivers with significant levels of mental distress from those with low distress^36,37^. Despite its relative novelty, the DASS-8 and its subscales have been used as criterion variables in many studies recruiting patient and healthy populations. They predicted decreased sexual satisfaction in women with endometriosis^39^, decreased academic achievement in university student^40^, psychotic-like experiences^41^ and reduced resilience in adolescents^42^ as well as disordered eating^43^, cyber-victimization, severe insomnia^44^, internal shame^45^, irritable temperament, feeling less valued by others, perceived dark future^46,47^, expressive suppression, low cognitive reappraisal, poor social support^48^, and self-perceived temporal perspectives (the past negative, present fatalistic, present hedonistic dimensions)^49^ in community-dwelling adults. DASS-8 scores also mediated the effect of exposure to media violence on all subtypes of aggression^50^ as well as the effect of mature religiosity on problem-focused disengagement at all levels of social support^51^.

Although all the DASS measures are not diagnostic^26,38^, people with high DASS scores embrace high risk of psychiatric morbidity, which may be ascertained in sophisticated clinician-facilitated diagnostic investigations. Hence, statistically determined DASS cutoff scores may improve the practicality of applying these symptom measures in clinical settings to highlight the presence of clinically meaningful anxiety and/or depression^23^. While the total score of the DASS measures demonstrates good convergent validity as a screener of distress^23,25,27,45^, lack of established cutoff scores for identifying populations with clinically significant levels of depression/anxiety is a major flaw associated with these measures^23,25^. The developers suggested that the total subscale scores, which range from 0 to 42 can reflect a range of conventional symptom severity from normal (below 9, 7, and 14) to severe (above 27, 19, and 33), for the symptoms of depression, anxiety, and stress, respectively^52,53^. Nonetheless, cutoffs of the DASS are used inconsistently: Lee et al. used only two cutoffs (normal and ≥ mild) ^53^, Cao et al. used scores ≥ 5, ≥ 4, and ≥ 8 to flag clinical depression, anxiety, and stress, respectively^52,54^ while cutoffs ≥ 1 of the three subscales were used to predict common mental disorders in post-partum women in Malawi^55^—a low score that commonly occurs in people with no mental problems despite high sensitivity and specificity reported in that study. Studies investigating cutoffs of the total scale are also inconsistent. In Vietnamese women with mood disorders, the DASS-21 and combinations of its subscales (anxiety with stress or anxiety with depression) better predicted mixed mood disorders without differentiating anxiety from depressive disorders at cutoffs ranging between 24 and 34^56^. Cutoffs of 32, 9, 7, and 14 were reported for the DASS-21 and its subscales in Chinese patients with traumatic brain injury^57^. Especially, DASS-21 cutoffs varied by gender; 14 and 17 cutoffs were reported for differentiating male and female adolescents with clinical distress^27^. DASS-21 scores ≥ 10 indicated that the prevalence of postpartum depression in women with breast cancer was 23%: 3% lower than that revealed by Structured Clinical Interview for DSM-IV (SCID) criteria and Beck Depression Inventory^58^. Research documents significant discrepancies in the prevalence of clinically significant mood disorders according to the cutoff of the DASS-21 and the DASS-Youth version, suggesting a need to alter cutoffs of the DASS-21^28^. Moreover, sensitivity analysis indicates that most items on the anxiety dimension are not significant in identifying the presence of anxiety disorders as portrayed by SCID in patients with brain injury^57^ or women with mixed mood disorders^56^. Likewise, co-calibration of the anxiety dimension against similar measures (Hospital Anxiety and Depression-Anxiety subscale and Generalized Anxiety Disorder scale-7) in a sample of cancer patients uncovered variations in the intensity of anxiety symptoms on the three measures (the DASS-anxiety subscale had a cutoff of 3 with unacceptable values of fit indices)^59^. Exploratory bifactor structure of the DASS-21 (Jennrich and Bentler vs Schmid–Leiman) showed weak factorial validity of the anxiety and stress subscales^22,60^, prompting the researchers to report only cutoffs of the DASS-21 (at a score of 16) and the depression subscale (two or more cutoffs) for detecting mood disorders^60^. Wide support for the bifactor structure of the DASS measures, with a general distress factor accounting for most of the variance and trivial contribution of its dimensions, implies that scale total scores reflect a unidimensional factor of negative emotions, which accounts for the overlap between depression and anxiety^22–24^. In line, theoretical backgrounds widely accepted for the treatment of mood disorders postulate that a superordinate temperamental trait of negative affect exists in both anxiety and depression, while lack of positive affect distinguishes unipolar depression from anxiety disorders^60^. Therefore, a well-validated, meaningful single cutoff of briefer versions of the DASS measure may support effective screening of psychological distress in large populations^23,24,28^, given their support for higher response rates^22^. To our knowledge, none of the shortened versions of the DASS-21 has been subjected to statistical investigations of its cutoffs except for the DASS-8^61^. Nonetheless, data were outdated (collected nine years before the study), and all predictors were examined by self-reported single-item questions, necessitating further validation of its cutoff^62–65^. Consequently, this study aimed to investigate cutoffs of the DASS-8 among dementia family caregivers basing its diagnostic potentials on predictors of caregiving burden and loneliness—common psychological problems closely associated with mental distress. Given the ominous effects of caregiving burden and loneliness, the resulting cutoff scores may be helpful for identifying caregivers vulnerable to psychiatric morbidity and in need of clinical help.

For a single measure, arbitrary cutoffs may result from arbitrary statistical methodologies, ranging from measures of variable distribution (e.g., means and medians) to machine learning models^63^. Clustering, an unsupervised multi-step learning technique, divides data into distinct groups with a high degree of inter-group similarity based on uncovered characteristics. In the initial stages, it detects and generates groups through a sequential approach, which pre-clusters cases and/or variables according to a distance measure that defines dense regions in the analyzed attribute-space. Then, it uses a probabilistic approach to statistically select and merge pre-clusters stepwise until the optimal subgroup model is determined, resulting in few non-overlapping groups^62,64,66^. Receiver operating characteristic (ROC) curve analysis is a valuable computational method, which aids in evaluating and comparing the diagnostic or prognostic accuracies of quantitative tests by determining clinically relevant cutoff scores—when a specific outcome has two possible endpoints, it classifies people as ‘‘positive’’ for this outcome^63,64^. ROC curve visualizes the diagnostic/prognostic possibilities of respective measures prior to categorization. It plots all potential cutoffs of binary tests that can be made from the continuously distributed values, with each value generating a point on a curve that stars at coordinates X0;Y0, goes vertically up the Y-axis and then horizontally across to coordinates X1;Y1^63^. The relationship between true and false positives is displayed, with true positivity (sensitivity) plotted against false positivity (1-specificity) for a chosen clinically relevant binary outcome on the curve^65^. Accurate cutoffs are noted by deviation of the ROC curve closer to the ‘‘optimal’’ upper-left handed corner, with larger area under the ROC curve (AUC)^60,63,65^. The diagnostic performance of a test variable is decided based on values of the AUC, sensitivity, specificity, and Youden index (J: (sensitivity + specificity) – 1)^65^. They should be closer to 1 in ideal tests, with a narrow 95% confidence interval (CI) for AUC values, which are persistently < 0.05. On the contrary, ROC curves run diagonally and have low fit indices in equally random tests, which comprise variables with no diagnostic/prognostic values^63,64^. Decision trees are machine learning techniques that set tree-like models that classify data into categories according to the target variables by exploring hidden patterns within the data. The model is inversely created with the root at the top with many branches downwards determined by a series of splits (internal nodes) based on conditions defined for the predictor variables. Branches, which end with child nodes (leaves), determine a decision. Criteria for the stop of tree growth may be chosen as either the longest path length from the root to a child node or by selecting a minimum number of training inputs for each child node^62,64^. The DASS-8 was tested through the three techniques in the current analysis.

## Material and methods

### Study design, participants, and procedure

This cross-sectional instrumental study comprised a convenience sample of 571 dementia family caregivers (mean age = 53 ± 12, range = 24–89 years) from northern Italy (74.4%) and southern Switzerland (25.6%). Caregivers were primarily females (n = 466, 81.6%) and adult children of care recipients (n = 410, 71.8%). Half the caregivers were employed (part-time or full time: n = 283, 49.6%), and most caregivers attained upper secondary education (n = 322, 56.4%). Care recipients had primarily Alzheimer’s disease (n = 316, 55.3%) and were dependent for activities of activities of daily living (ADL; n = 455, 79.7%). More details on the characteristics of the participants are available elsewhere^36,67^. Data were collected between May 25th and June 25th, 2020, through an online survey conducted via RedCap. The participants signed a digital informed consent before completing the test battery.

### Measures

The questionnaire administered to the participants was in the Italian language, and it contained questions about their sociodemographic characteristics (e.g., age, gender, education, etc.), relationship with care recipients (adult children or spouses), receiving help with caregiving (yes or no e.g., from another family member, neighbor, or a paid caregiver), type of dementia, and the level of care recipient dependency for ADL (autonomous or not autonomous). The test battery comprised three measures:

1. The Depression Anxiety Stress Scale-8 (DASS-8) comprises 8 items in three subscales, which capture depression, anxiety, and stress symptoms. The stress subscale comprises two items “e.g., I was using a lot of my mental energy” while each of the depression and anxiety subscales comprises three items “e.g., I felt that I had nothing to look forward” and “e.g., I felt close to panic”, respectively. The respondents rate the frequency of endorsing the items of the DASS-8 over the past week on a scale of four response categories ranging from 0 (did not apply to me at all) to 3 (applied to me very much or most of the time). Our respondents were instructed to rate their symptoms over the last month for better detection of their distress during the peak of the COVID-19 pandemic (the time of data collection)^67^. The total scores of the DASS-8 and its depression, anxiety, and stress subscales range between 0 (all the measures) and 24, 9, 9, and 6, respectively^26,38^. Its reliability in this study is excellent (coefficient alpha = 0.93).
2. The Zarit Burden Interview (ZBI)^68^ comprises 22 items, which capture caregiving burnout: adverse effects of caring for a patient with dementia on caregivers’ physical and mental health, quality of life, relationships, and finance^14^. Example items include “feel strained, feel angry, social life suffering, financial stress, health affected, and lack privacy”. Respondents rate the frequency of endorsing items of the ZBI on a five-point Likert scale ranging from 0 (never) to 4 (nearly always). The total scores of the ZBI range between 0 and 88. Higher scores reflect greater burden^68,69^. Its reliability in this study is excellent (coefficient alpha = 0.94).
3. The Three-Item Loneliness Scale is a brief measure, which has been developed as a refinement of the University of California, Los Angeles, Loneliness Scale-version (UCLALS), herein referred to as UCLALS3^68,70^. It comprises three items “e.g., how often do you feel that you lack companionship”. Items are rated on a three-point scale ranging from 1 (hardly ever) to 3 (often). The total scores of the UCLALS3 range between 3 and 9. Higher scores indicate greater loneliness^70^. Its reliability in this study is very good (coefficient alpha = 0.87).

### Statistical analysis

Quartile scores of the DASS-8 were calculated to determine its initial cutoff scores. In K-means clustering analysis, the participants were located into non-overlapping clusters based on the scores of the DASS-8 and its subscales^37^. Decision tree and ROC analyses were conducted using the continuous scores of the DASS-8 to predict caregiving burden and loneliness, which were both used as categorical variables. ZBI categories were created based on the cutoff (48) reported for predicting depression and anxiety among family caregivers of patients with schizophrenia^71^. Because we found no cutoff score of the UCLALS3, we created its categorical form using the median (7), which can be acceptable for setting thresholds that reflect variable distribution^65^. This method does not eliminate uncertainty in time-to-event endpoints of the outcome of interest^63^ unlike advanced techniques (e.g., ROC analysis and decision trees), which employ optimal separation between groups concerning some outcomes. However, it was not possible to use these techniques to examine the UCLALS3 because of lack of predictor variables required for these tests^62,63^, which also fall beyond the scope of this study. In ROC analysis, DASS-8 cutoffs were selected based on fit indices discussed above^64,71,72^. In decision tree analysis, we employed the classification and regression (CRT) growing method with the validation method of random assignment of cases into training and test samples (70 and 30% of cases, respectively)^64^. The test sample acts as a source of prior probabilities of group membership. Prior probabilities are estimates of the overall relative frequency for each category of the dependent variable prior to knowing anything about the values of the independent (predictor) variables. They aid in minimizing tree growth caused by data in samples that are not representative of the entire population^73^.

Apart from cutoff score analyses, a general path model was used to investigate whether the DASS-8 can reflect burnout and distress. Predictors in the model comprised the ZBI, UCLALS3, and sociodemographic characteristics of the respondents. We trimmed nonsignificant paths. Model fit was considered good based on a non-significant χ^2^, along with discrepancy divided by degree of freedom (CMIN/DF) < 3, Comparative Fit Index (CFI) and Tucker–Lewis Index (TLI) > 0.95, standardized root-mean-square residual (SRMR), and root mean square error of approximation (RMSEA) < 0.06^74,75^. The analyses were performed in IBM SPSS Statistics (version 28) and IBM Amos Statistics (version 24). Significance was considered at a probability < 0.05 in two-tailed tests.

### Ethics approval and consent to participate

The study was conducted according to the Declaration of Helsinki. The protocol for data collection has been approved by the Italian and Swiss Cantonal ethics committee^67^, and no further ethical approval was obtained because the study does not fall within the scope of Art 2 and Art 3 of the law on human research. The dataset generated is shared under the terms of Creative Common License (CC BY 4.0)^76^. Accordingly, no ethical approval was attained for the current secondary analysis.

## Results

**Figure 1:**
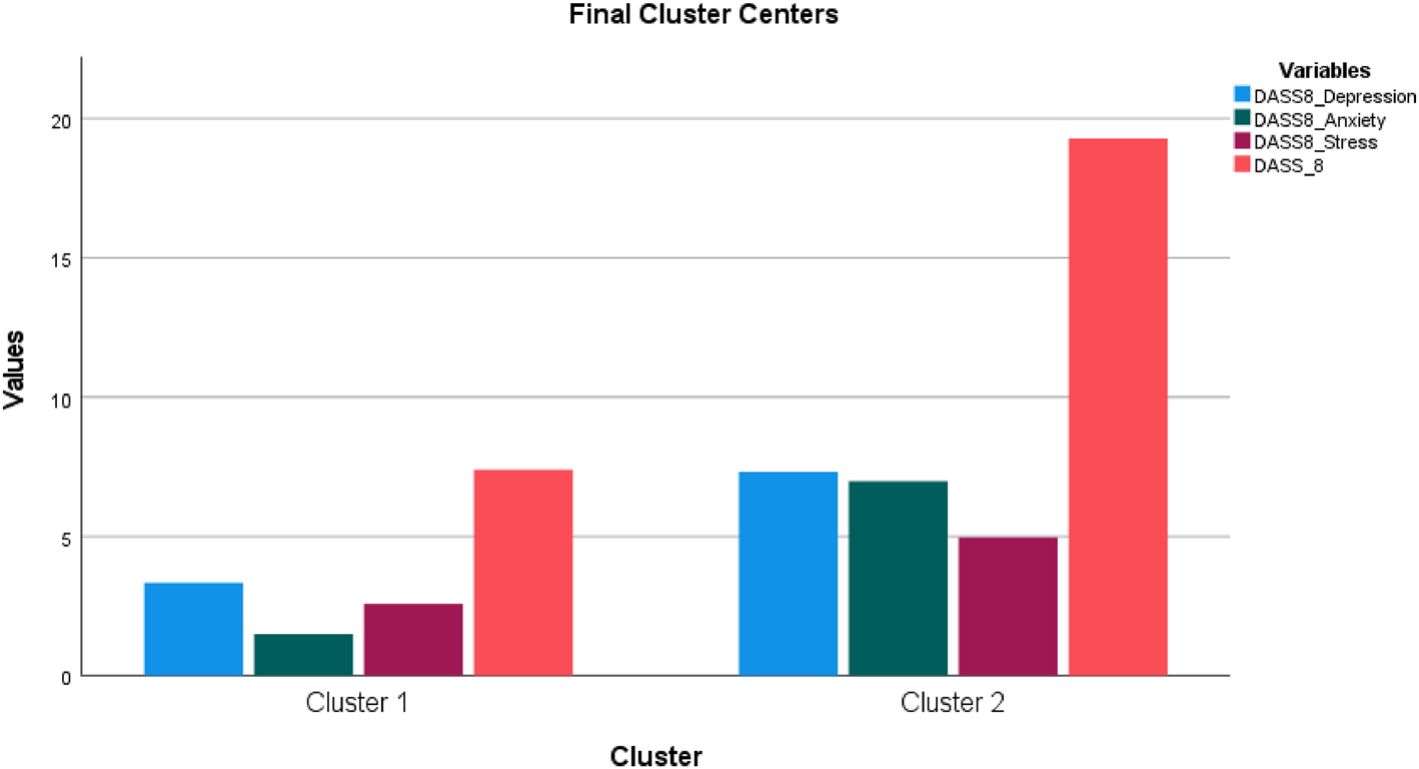
K-means clusters of dementia family caregivers according to the scores of the Depression Anxiety Stress Scale 8 (DASS-8) and its subscales.

The mean scores of the DASS-8, ZBI, and UCLALS3 were 13.6 ± 6.9, 54.3 ± 18.3, and 6.8 ± 2.1, respectively. The quartile scores of the DASS-8 were 8.0, 15.0, and 19.0. Clustering analysis classified caregivers into two clusters reflecting low (n = 271) and high (n = 300) levels of distress. Cutoff scores of the DASS-8, depression, anxiety, and stress subscales that were used to locate the participants into clusters were 19.0, 7.0, 7.0, and 5.0, respectively (Fig. 1). ANOVA test shows that the differences between clusters were significantly maximized (F(212) = 536.83, 246.19, 316.57, and 227.89; all p values < 0.001).

**Figure 2:**
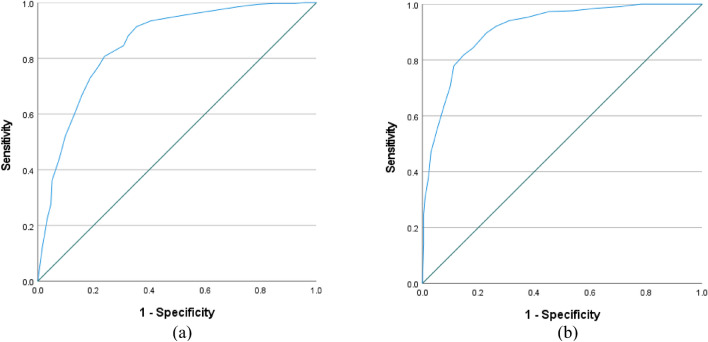
Receiver-operating characteristic (ROC) curve using the scores of the Depression Anxiety Stress Scale 8 (DASS-8) to classify dementia family caregivers according to their levels of caregiving burden (Zarit Burden Interview (ZBI), a) and loneliness (University of California, Los Angeles, Loneliness Scale-version 3 (UCLALS3), b).

Based on the ZBI cutoff, ROC-curve criteria (AUC = 0.85, SE = 0.02, p < 0.001, 95% CI 0.82 to 0.88, sensitivity = 0.81, specificity = 0.76, Youden index = 0.57) show that DASS-8 cutoff of 12.5 can discriminate caregivers with high and low caregiving burden. Based on the UCLALS3 cutoff, ROC-curve criteria (AUC = 0.91, SE = 0.01, p < 0.001, 95% CI 0.89 to 0.94, sensitivity = 0.78, specificity = 0.89, Youden index = 0.67) show that DASS-8 cutoff of 14.5 can discriminate caregivers with high and low loneliness (Fig. 2).

**Figure 3:**
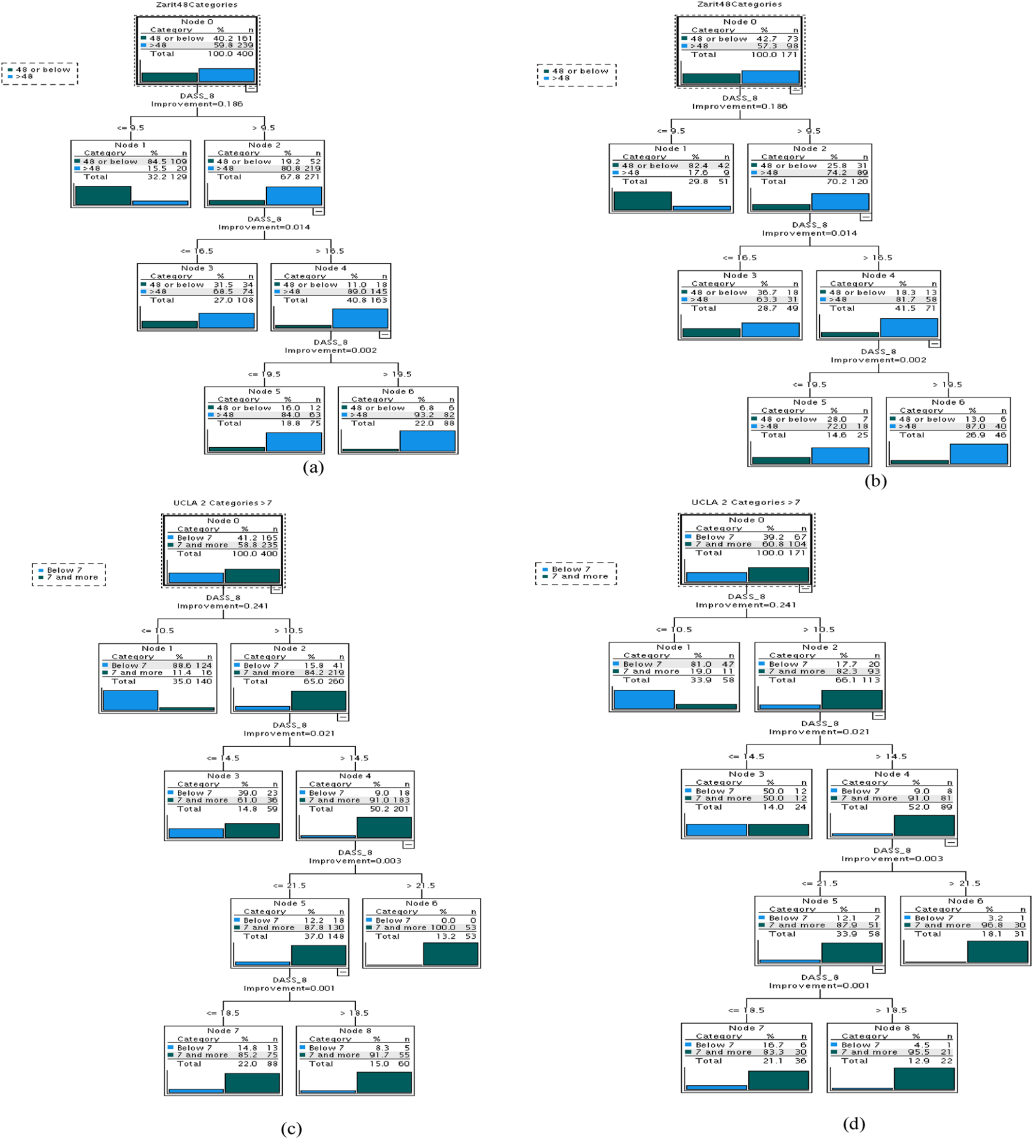
Decision tree models using the Depression Anxiety Stress Scale 8 (DASS-8) to predict caregiving burden (Zarit Burden Interview (ZBI), **a**: training sample, **b**: test sample) and loneliness (University of California, Los Angeles, Loneliness Scale-version 3 (UCLALS3), **c**: training sample, **d**: test sample).

Decision tree analysis indicated that 9.5 DASS-8 cutoff may distinguish between caregivers with high and low burden (Fig. 3a,b). However, higher cutoffs (16.5 and 19) demonstrated a greater probability of prediction. Consistent with ROC analysis, decision modeling indicated that 14.5 DASS-8 cutoff may efficiently predict loneliness (Fig. 3c,d). However, the probability of prediction was greater at higher scores (e.g., 18.5 and 21.5).

To determine a single optimal cutoff score of the DASS-8, cutoffs obtained from all analyses were ordered ascendingly (9.5, 12.5, 14.5, 14.5, 15.0, 19.0). The value in the middle was chosen (14.5) as an optimal cutoff in this sample.

**Figure 4:**
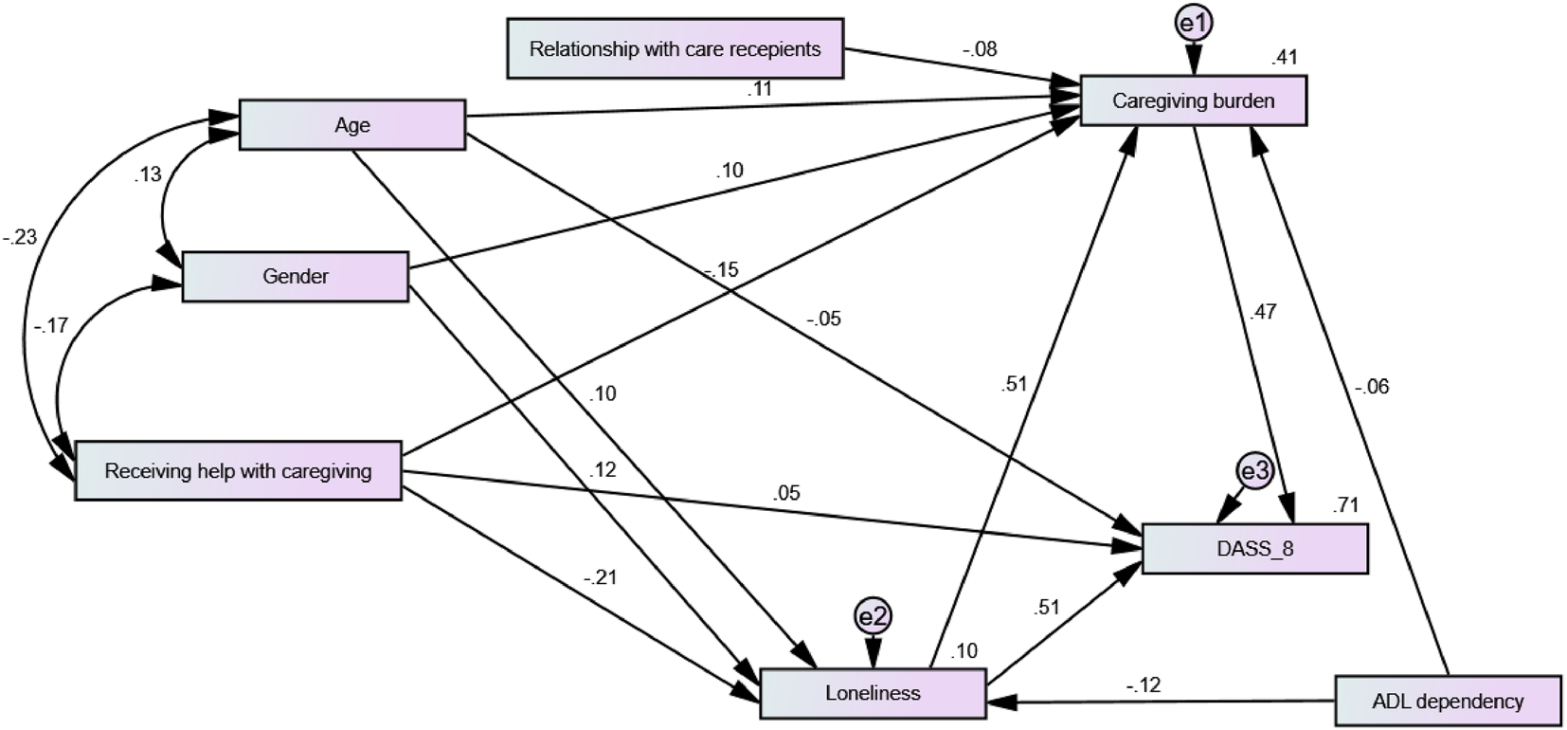
Path analysis model predicting scores of the Depression Anxiety Stress Scale 8 (DASS-8) based on the continuous scores of the Zarit Burden Interview (ZBI), the University of California, Los Angeles, Loneliness Scale-version 3 (UCLALS3), and the sociodemographic characteristics of dementia family caregivers.

The path model expressed excellent fit (χ^2^ = 11.89, df = 11, p = 0.3, CMIN/DF = 1.08, CFI = 0.999, TLI = 0.998, RMSEA = 0.012, RMSEA 95% CI 0.000 to 0.046, SRMR = 0.026). It predicted 71% of the variance in psychological distress. Because non-significant paths were trimmed, all direct relations in Fig. 4 are significant. Loneliness and caregiving burden predicted 51 and 47% of the variance in the DASS-8, respectively (p values < 0.001). Loneliness predicted 51% of the variance in caregiving burden, and it expressed an indirect effect on distress through caregiving burden (β = 0.238, 95% CI 0.204 to 0.278, p = 0.011).

Age directly predicted loneliness and caregiving burden (p = 0.001 and 0.020). While its direct effect on distress was negative (p = 0.036), it expressed a positive indirect effect on distress through caregiving burden (β = 0.120, 95% CI 0.057 to 0.173, p = 0.012). Gender was a strong direct predictor of loneliness and caregiving burden (both p values = 0.004). Mann Whitney U test revealed greater levels of loneliness (U = 17,952.0, z = − 4.21, p < 0.001) and caregiving burden (U = 15,431.5, z = -5.81, p < 0.001) among females. Although gender had no direct effect on distress, it exerted significant positive indirect effects on distress through loneliness (β = 0.131, 95% CI 0.086 to 0.185, p = 0.012) and caregiving burden (β = 0.059, 95% CI 0.031 to 0.086, p = 0.020). Receiving help with caregiving negatively predicted caregiving burden and loneliness (p values < 0.001). It positively predicted distress (p = 0.034), but its indirect effects on caregiving burden and distress were negative (β = − 0.107 and − 0.228, 95% CI − 0.140 to − 0.068 and − 0.281 to − 0.161, p values = 0.012).

ADL dependency negatively predicted caregiving burden and loneliness (Fig. 4, p = 0.048 and 0.002) without a direct effect on distress. It exerted significant indirect effects on distress (β = − 0.120, 95% CI − 0.189 to − 0.066, p = 0.006) and caregiving burden (β = − 0.062, 95% CI − 0.106 to − 0.029, p = 0.008). Distress scores were significantly greater among those caring for ADL-dependent patients compared with those who cared for ADL-autonomous patients^36^. Caregivers’ relationship with care recipients negatively predicted caregiving burden (Fig. 4, p = 0.019). Spouses had greater caregiving burden (U = 17,172.5, z = − 4.81, p = 0.001) and loneliness scores (U = 18,013.5, z = − 4.31, p = 0.001) compared with adult-children. However, distress scores were significantly greater among adult children relative to spouses^36^.

## Discussion

Caring for demanding, chronic conditions such as dementia can deplete caregivers’ emotional resources, resulting in poor mental and physical health^10,13^. Quick screening for distress may be a cost-effective method for early detection and treatment of psycho-pathogenicity among caregivers^7,8,16^. In practice, cutoffs of continuous measures represent a straightforward method for classifying individuals as “positive or high risk” and “negative or low risk” for specific conditions. A single study testing DASS-8 cutoffs revealed an array of scores, which predicted miscellaneous outcomes in women with chronic pelvic pain^61^. To provide unbiased, statistically-established optimal cutoff of the DASS-8, the current study comes in concordance with calls for testing measures using several robust strategies^62–64^ in different independent datasets (herein dementia caregivers) to minimize risk bias and type-I error^65^. Many cutoffs of the DASS-8 emerged in our analyses (clustering, ROC analysis, and decision tree). Referring to the literature^64,65^, a single optimal cutoff was defined by choosing the median of all the resulting cutoffs. Accordingly, caregivers with DASS-8 scores above 14.5 should undergo advanced investigations for psychiatric problems.

Previous research has examined cutoffs of the DASS-21 in different populations. Given the dearth of studies investigating cutoffs of the DASS-8, herein, we take advantage of the fact that AUC values as general calculations of the global diagnostic accuracy of measures may aid in general comparisons of two or more diagnostic tests^60^. Our values of AUC, Youden index, sensitivity, and specificity indices show comparable or even greater global diagnostic accuracy of the cutoff of the DASS-8 relative to the parent scale—suggesting that not too many false-negative or false positive cases may emerge at a DASS-8 cutoff of 14.5. For example, DASS-21 cutoffs of 10, 16, and 34 (AUC values = 0.81, 0.67, and 0.81) predicted mental health problems in Arab women with breast cancer^58^, healthy students from the USA^60^, and women with mood disorders from rural Vietnam^56^, respectively. In this sense, our results supplement existing knowledge, which may employ the cutoff of the DASS-21 as a beneficial component of an actuarial decision-making strategy within the context of stepped healthcare delivery system—by defining individuals in need for additional psychiatric evaluations^60^.

Unlike previously reported cutoff scores for the DASS-8, clustering analysis defining groups based on solo differences in the DASS-8 and its subscales, in the absence of external predictor variables, distinguished two clusters of caregivers based on a DASS-8 score of 19. This score corresponds to the third quartile score of the DASS-8. However, different cutoffs appeared in tests using burnout and loneliness as outcome measures. These scores are consistent with previously recorded cutoffs of the DASS-8. Particularly, ROC analysis revealed significant burnout and loneliness at DASS-8 cutoffs of 12.5 and 14.5 with AUC values of 0.85 and 0.92, respectively. The same DASS-8 cutoffs, in order, previously predicted intake of over-the-counter medicine and high vs low distress in women with pelvic pain^61^. The present values of AUC and Youden index were greater in the model predicting loneliness—denoting greater diagnostic potentials of the DASS-8 14.5 than 12.5 cutoff^64^. This score was closer to the median of the DASS-8 (15.0). Median scores represent a plain high vs low distress threshold^65^. Indeed, the median of the DASS-21 successfully differentiated women diagnosed with depression or anxiety disorders from healthy counterparts^56^. Decision tree modeling shows that the DASS-8 at a score of 9.5 may discriminate people with high and low caregiving burden. The same DASS-8 score predicted self-reported psychiatric comorbidity in women with chronic pelvic pain^61^. Interestingly, decision tree and ROC analyses predicting loneliness produced DASS-8 cutoffs with similar values, implying robust applicability of the finally defined cutoff^64^. Given greater diagnostic accuracy of the narrow range of the DASS-8 cutoffs seen in the present and previous investigations relative to the considerably wide range of cutoffs (10 to 34) of the DASS-21, the DASS-8 may be more acceptable as a simple measure of distress symptoms.

It is worth mentioning that the probability of prediction in both decision tree models increased at higher scores (16.5 and 19 for burnout; 18.5 and 21.5 for loneliness). Evidently, those higher scores were closer to the score produced in clustering analysis and the third DASS-8 quartile score. Thus, they may correspond to higher degrees of severity (severe or extremely severe distress). Similarly, scores below the first quartile may reflect normal/mild distress^63^. However, this is not the scope of this study, which aims to determine an optimal cutoff: a single diagnostic score that can distinguish subjects as high and low on the measurement at hand without interest in defining different degrees of severity (e.g., low, mild, moderate, high, severe/extremely severe)^64^. Further investigations may examine multiple levels of distress captured by the DASS-8.

The DASS-8 is a brief measure, which can be easily self-applied to elicit a greater response rate. Its ability to predict higher loneliness and caregiving burden signifies that higher DASS-8 scores may also reflect greater loneliness and burnout, even without measuring these outcomes directly. To test this hypothesis, we ran a path model to see if DASS-8 scores reflect the distressing psychological experiences of loneliness and caregiving burden among dementia caregivers. As expected, greater distress appeared as an outcome of higher scores of loneliness and caregiving burden. The model included caregivers’ sociodemographic characteristics, which operated interweavingly to affect loneliness, burnout, and distress. Not surprisingly, care recipients’ ADL dependency was a source of greater caregivers’ distress. Meanwhile, receiving help with caregiving was associated with a significant direct and indirect reduction in caregiving burden along with a direct increase and indirect decrease in distress scores. Group comparisons revealed greater loneliness (U = 27,758.5, z = − 6.18, p = 0.001) and lower caregiving burden (U = 24,140.5, z = − 7.93, p = 0.001) among those who received help than those who did not. Given the mediating role of caregiving burden and loneliness on the effect of receiving help on distress, it seems that lonely caregivers’ tendency to seek assistance with caregiving may reduce their burden and indirectly reduce their distress, but it does not totally free them from mental distress. This might be attributed to doing more effort to secure the availability of an alternative caregiver during COVID-19’s periods of lockdowns, which witnessed extensive social isolation, global economic recession, dramatic loss of employment, and rise in prices^36,77^. The cost of respite care, which on average exceeds one quarter of the income of family caregivers, is another documented source of distress^9^. Likewise, depression in caregivers of people with Alzheimer's disease is associated with increased numbers of caregivers^11^. Same as in caregivers of people with Alzheimer's disease, being a spouse was associated with greater loneliness and burnout^11^. Possibly because of their advanced age, spouses had extended duration of caregiving (r = 0.257, p < 0.001), which is further associated with caregiving burden, loneliness, and distress (r = 0.350, 0.290, and 0.393; p values < 0.001). This coincides with higher levels of loneliness and burnout among older participants (Fig. 4). In line, caregivers of people with Alzheimer's disease who were caregivers for longer durations displayed elevated levels of caregiving burden and anxiety^11^. Loneliness expressed a significant indirect positive effect through caregiving on distress. Research suggests a bidirectional relationship between loneliness and negative emotional conditions (major depression and generalized anxiety disorders, sadness, and stress)^78,79^. However, longitudinal data show that elevated levels of loneliness predict more severe symptoms of social anxiety and depression over time^78,79^. Taken together, our findings imply that younger caregivers (who were predominantly middle-aged) are less prone to distress as a function of their own capacity to provide care more efficiently and keep themselves less lonely than older counterparts. In support, research reports higher caregiving burden and loneliness in older adults relative to middle-aged caregivers^15^. Although gender had no direct effect on distress, females displayed greater caregiving burden and loneliness. Gender indirectly increased distress through caregiving burden and loneliness. In summary, high distress scores reflect greater loneliness, caregiving burden, ADL-dependency, being involved in care for relatively long durations, and greater need for help with caregiving, especially among older, female, and spouse caregivers. These specific groups of caregivers have been previously reported to struggle with higher levels of distress^7,80–82^. Thanks to its brevity, use of the DASS-8 for frequent screening may allow practitioners to be more specific and effective in detecting caregiver distress, especially within groups with identified risk for burden and loneliness.

One merit of this study is analyzing publicly accessible data through robust techniques (ROC analysis and decision modeling) to determine an optimal cutoff of the DASS-8 that may effectively predict critical mental health-related problems (caregiving burden and loneliness) in a highly vulnerable population. Decision tree analysis employed a validation method: 70 and 30% of the participants were simultaneously located into training and test samples, which permits accounting for variations related to the data in the samples^64^. Our study has clinical and research implications. The results show that distress in dementia caregivers is a direct and indirect function of loneliness and caregiving burden, along with their interaction with a variety of demographic and clinical variables. Therefore, higher DASS-8 scores (14.5 and above) may reflect loneliness, burnout, ADL-dependency, and need for help with caregiving, especially among female, older, and spouse caregivers who represent targets for the detection and treatment of psychiatric morbidities. The results should be interpreted with caution, because the study has limitations. Online surveying, convenience sampling, and cross-sectional design entail risks for selection and reporting bias. Because comorbidities are associated with lowered resilience and increased proneness to psycho-pathogenicity^77^, our respondents should have been screened for mental problems (e.g., mixed mood disorders) through clinician-based interviews or through other measures of distress, depression, and anxiety to validate the cutoff scores. However, this was not possible since the analyses used a public dataset. Accordingly, lack of assessment of the history of mental and physical illness in the sample may confound the results. Alternatively, the analysis involved already available measures of closely linked constructs (burnout and loneliness)^5,15^, which may lend some support to the credibility of our results, albeit they are still not doubt-free. Moreover, loneliness and burnout were included in path model as observed variables. Assuming absence of measurement error does not provide closer-to-reality results as when latent variables are tested. Additionally, the direction of relations revealed in path analysis may not reflect the reality given the cross-sectional nature of the data. Data were collected during the peak of the COVID-19 from a border area involving north Italy and south Switzerland. In the meantime, our variables (e.g., loneliness and distress) occur at greater rates in individualistic cultures (e.g., Europe), which focus primary on individuals' interests rather than group norms and social connections^15,38^. Therefore, data and characteristics of our subjects may diverge from those found in other areas and time points. The questionnaires were addressed in Italian assuming that all the respondents could fluently speak Italian, but this assumption was not proved by inquiring about the level of fluency. Consequently, further examinations in populations from different cultural backgrounds may ensure the reliability of the reported cutoffs.

## Conclusion

Different statistical methods indicate usefulness of the DASS-8 at a score of 14.5 for predicting high burnout and loneliness among dementia caregivers. Referring caregivers with higher DASS-8 score, especially females, elderly, and spouses, for advanced assessment and treatment of psychiatric comorbidities may promote their wellbeing and care continuity. Replications of the study in other countries may confirm the findings.

## Data Availability

The dataset supporting the conclusions of this article is available in Zenodo repository, [https://zenodo.org/record/4748652#.YdbwiWhBw2w]^76^, and also the datasets used and/or analyzed during the current study are available from the corresponding author on reasonable request.
